# A Scoping Review of Corticosterone-Induced Changes in Ionotropic Glutamate Receptor Levels and Localization in the Rodent Brain: Implications for the Auditory System

**DOI:** 10.3390/brainsci15020110

**Published:** 2025-01-24

**Authors:** Elsa Edlund, Ewa Domarecka, Heidi Olze, Agnieszka Szczepek

**Affiliations:** 1 Department of Otorhinolaryngology, Head and Neck Surgery, Charité-Universitätsmedizin Berlin, Corporate Member of Freie Universität Berlin and Humboldt-Universität zu Berlin, 10117 Berlin, Germany; elsa.edlund@charite.de (E.E.); ewa.domarecka@charite.de (E.D.); heidi.olze@charite.de (H.O.); 2 Faculty of Medicine and Health Sciences, University of Zielona Góra, 65-046 Zielona Góra, Poland

**Keywords:** AMPA, AMPA receptor, GluR, glutamate receptor, stress, corticosterone, HPA-axis

## Abstract

Background: The ionotropic glutamate receptor AMPA (AMPAR) mediates fast excitatory synaptic transmission and regulates synaptic strength in various parts of the CNS. Emotional challenges can affect these processes by influencing AMPAR levels and localization via stress hormones, resulting, e.g., in behavioral changes. AMPARs are essential for auditory processing, but their response to stress hormones in the central or peripheral auditory system remains poorly understood. Therefore, this scoping review examines the effects of corticosterone (CORT), a primary stress hormone in rodents, on AMPA receptor levels and localization in the rodent nervous system and considers potential implications for the auditory system. Methods: We systematically searched PubMed, Web of Science, and OVID EMBASE using MeSH terms related to AMPA receptors and corticosterone. Studies were screened based on predefined inclusion criteria, including original research published in English that focused on AMPA receptor subunits (e.g., GluR1-4, GluA1-4, Gria1-4). Of 288 articles screened, 17 met the criteria for final analysis. Results: No reports were found regarding CORT action in the auditory system. Three main experimental models used in the included research were identified: neuronal cultures, isolated tissue cultures, and animal models. Generally, short-term CORT exposure increases AMPAR surface localization and mobility in neuronal cultures, especially in the hippocampus and prefrontal cortex. However, results from animal models were inconsistent due to variations in experimental design and other factors. The isolated tissue study did not provide sufficient data for clear conclusions. Conclusions: Variability in experimental models limits our ability to draw definitive conclusions about the effects of CORT on AMPARs across different regions of the nervous system. The differences in live animal studies highlight the need for standardized methods and reporting. Since AMPARs play a crucial role in auditory processing, CORT-induced changes in neuronal cultures may occur in the auditory system. Further research is needed to explore the specific responses of AMPAR subunits and how stress hormones may influence auditory disorders, which could help identify potential treatment strategies.

## 1. Introduction

### 1.1. Stress and the Auditory System

For nearly two centuries, clinical observations have suggested a relationship between emotional stress and auditory disturbances, particularly tinnitus, characterized by the perception of phantom sounds without external stimuli [1,2]. As early as 1841, John Curtis reported in The Lancet that patients experienced tinnitus following distressing life events [3]. Recently, McKenna suggested that awareness of this connection may extend even further back, noting that the ancient Greek poet Sappho described a buzzing in the ears when overwhelmed by emotions—an early hint of the link between psychological state and auditory disturbances [4]. Modern epidemiological studies have since confirmed a significant association between stress and the incidence of tinnitus, comparable to the impact of noise exposure [5]. Tinnitus is notably more prevalent among individuals in high-stress occupations, and symptoms often worsen during increased stress [4,6,7].

Several studies during the SARS-CoV-2 pandemic noted that individuals with pre-existing tinnitus experienced a worsening of their condition due to elevated stress. Xia et al. found significant increases in anxiety among tinnitus patients in the first year of the pandemic compared to the previous year [8]. Schlee et al. reported that emotions like grief and frustration were linked to heightened tinnitus distress [9]. Beukes et al. noted that one-third of tinnitus patients experienced worsening symptoms due to pandemic-related concerns [10]. Erinc et al. found increased tinnitus-related complaints in those who contracted SARS-CoV-2, while Korkut and Altıntaş reported that over 80% of tinnitus patients felt their symptoms intensified during this time [11,12]. However, Fioretti et al. and Aazh et al. did not find significant changes in tinnitus severity linked to the virus or pandemic stress [13,14]. Finally, a systematic review has concluded that COVID-19 has negatively impacted tinnitus, suggesting that psychological stress can worsen the condition [15].

Research conducted on individuals with post-traumatic stress disorder (PTSD) shows that tinnitus symptoms are frequently observed in this population [16,17]. This finding underscores the connection between stress and tinnitus. The effectiveness of stress-management techniques in reducing tinnitus symptoms emphasizes the significant role that stress plays in developing this condition [16]. Additionally, tinnitus can act as a stressor, potentially creating a cycle where increased stress exacerbates tinnitus, which raises stress levels further [4].

There is a recognized connection between auditory pathways and the limbic system responsible for processing emotions. This relationship suggests that stress can affect emotional responses to sound, potentially leading to the onset or worsening of auditory disorders. Emotional stress has been associated with several conditions, including auditory hallucinations, sudden sensorineural hearing loss, Menière’s disease, hyperacusis, and misophonia [17,18,19]. Although the exact mechanisms are still unclear, a well-documented link exists between stress and auditory disturbances. Proposed theories indicate that the hypothalamic–pituitary–adrenal (HPA) axis may play a role in influencing neuroplasticity and glutamatergic neurotransmission [17].

Tinnitus is associated with disruptions in tonotopy, which refers to the spatial arrangement of frequency-specific neurons within the auditory pathways. Research on tinnitus patients has demonstrated altered tonotopic organization in the auditory cortex. Additionally, studies using animal models have suggested similar changes in the cochlea [20]. This tonotopic reorganization indicates the presence of underlying neural plasticity, which is the process by which the nervous system adapts to changes in sensory input. The mechanisms of neural plasticity are influenced by glutamatergic signaling, with AMPA receptors (AMPARs) playing a vital role in facilitating synaptic changes.

It is important to note that experimental stress can influence auditory thresholds in animals, as assessed by auditory brainstem responses [21,22]. The degree and nature of this modulation vary depending on the animal strain and the time elapsed after the stressor. However, the specific mechanisms underlying these changes have yet to be clarified.

### 1.2. Glutamate and the Auditory System

Glutamate, the primary excitatory neurotransmitter in the auditory system, interacts with several types of ionotropic glutamate receptors—AMPA, N-methyl-D-aspartate (NMDA), kainite—as well as metabotropic glutamate receptors (mGluRs). While NMDA receptors, kainate receptors, and mGluRs primarily modulate signaling pathways in the auditory system, AMPARs are responsible for direct glutamate transmission, enabling precise and fast auditory signal transmission [23].

**Figure 1:**
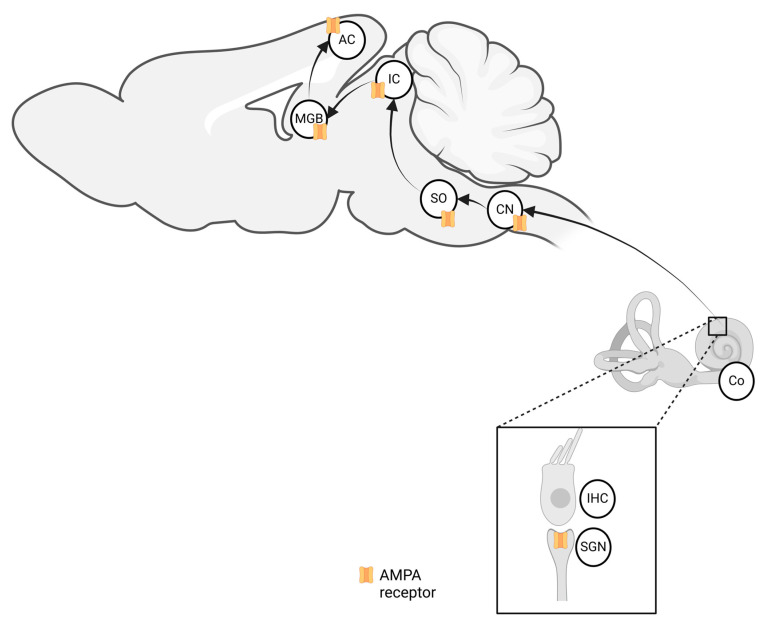
Schematic representation of the ascending auditory pathway in rodents, highlighting the key structures where AMPAR-mediated glutamatergic transmission occurs [26,27,28]. Legend: Co, cochlea; IHC, inner hair cells SGN; spiral ganglion neuron; CN, cochlear nucleus; SO, superior olive; IC, inferior colliculus; MGB, medial geniculate body; AC, auditory cortex. Created with Biorender.com (accessed on 21 January 2025).

Auditory processing depends on a fundamental process in the inner ear, where cochlear inner hair cells convert mechanical sound waves into neural signals by releasing glutamate. This glutamate activates post-synaptic α-amino-3-hydroxy-5-methyl-4-isoxazolepropionic acid receptors (AMPARs) on spiral ganglion neurons forming the auditory nerve. The signal then travels to the central nervous system for processing. This transmission is among the fastest in the nervous system and is essential for the temporal precision required in auditory perception. It relies on the rapid excitatory glutamate signaling mediated by AMPARs [24,25]. The auditory pathway involving AMPAR-mediated glutamatergic transmission is shown in Figure 1.

**Figure 2:**
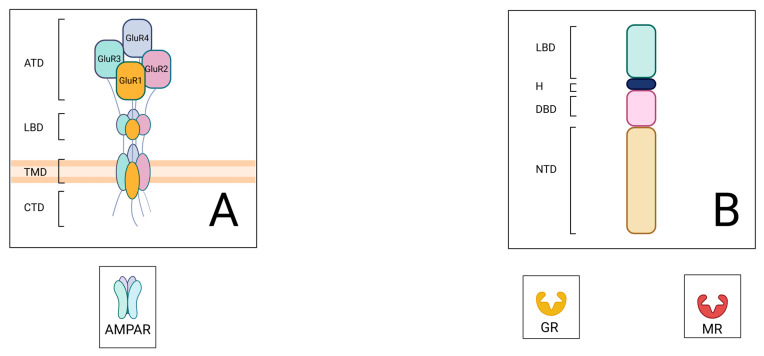
Schematic representation of the structures of AMPA and glucocorticoid receptors. (**A**) The heteromeric AMPA receptor is composed of four GluR subunits. The key components include the amino-terminal domain (ATD), ligand-binding domain (LBD), transmembrane domain (TMD), and C-terminal domain (CTD). AMPAR stands for AMPA receptor. (**B**) The general structure of nuclear receptors that can bind glucocorticoids consists of the ligand-binding domain (LBD), hinge region (H), DNA-binding domain (DBD), and N-terminal domain (NTD). GR refers to the glucocorticoid receptor, while MR refers to the mineralocorticoid receptor. Created with Biorender.com (accessed on 21 January 2025).

AMPARs are ionotropic receptors composed of four subunits, GluR1-4, which form heterotetramers [29,30]. The structural composition of AMPA receptors is characterized by three distinct domains: the extracellular amino-terminal domains, the ligand-binding domains, and the transmembrane domains (Figure 2) [31]. The AMPA subunits are critical in regulating glutamate-induced gating kinetics, ion channel permeation, and drug sensitivity. After their assembly in the endoplasmic reticulum, AMPARs are trafficked through the neuron’s secretory pathway to the plasma membrane of a dendritic spine. Receptor density is maintained at the synapse through lateral diffusion and constitutive recycling to support receptor plasticity and synaptic responsiveness [32]. Synaptic strength is dynamically regulated by the number and composition of AMPARs at excitatory synapses, allowing continuous adaptation to neuronal activity. Evidence suggests that the composition of the subunits GluR1-4 is critical for AMPAR functionality and trafficking and that the phosphorylation of specific subunits can further regulate the trafficking dynamics [32,33,34].

The precise mechanisms involved in the assembly, trafficking, and regulation of AMPA receptors (AMPARs) are still an active area of research. However, the dynamic regulation of synaptic strength is essential for their role in neuronal plasticity. Activity-dependent processes, such as the insertion and removal of AMPARs at synapses, are central to mechanisms like long-term potentiation (LTP) and long-term depression (LTD), which are fundamental for synaptic remodeling and adaptability [35]. One well-established factor influencing AMPAR-mediated plasticity is stress. For instance, studies by Krugers et al. [36] and Popoli et al. [37] demonstrate how stressors can affect neuronal plasticity by altering glutamatergic signaling through AMPARs.

### 1.3. Stress Responses and Neuroplasticity

Stress is a critical mechanism that prepares organisms for “fight or flight” responses. This adaptive response is regulated by the hypothalamic–pituitary–adrenal (HPA) axis. When exposed to stressors, the hypothalamus releases corticotropin-releasing hormone, which stimulates the anterior pituitary gland to secrete adrenocorticotropic hormone. This, in turn, prompts the adrenal glands to release corticosteroids [38]. In humans, the primary endogenous corticosteroid is cortisol, while in rodents, it is corticosterone.

Glucocorticosteroids exert their effects by binding to specific receptors, which are classified into two types: glucocorticoid receptors (encoded by the Nr3c1 gene in mice) and mineralocorticoid receptors (encoded by the Nr3c2 gene in mice) [39]. Both receptor types consist of four main regions: an N-terminal domain, a DNA-binding domain, a hinge region, and a ligand-binding domain (Figure 2). The DNA-binding domain is highly conserved and plays a critical role in the receptors’ function as transcription factors. This domain is also responsible for the dimerization or oligomerization of the receptors. In contrast, the N-terminal domain serves as a target for co-regulatory molecules.

Decades of research have established that the release of corticosteroids via the activation of the HPA axis influences neuroplasticity—the ability of the nervous system to change in response to stimuli by reorganizing its structure, functions, or connections. Alterations in synaptic AMPAR levels contribute to stress’s adaptive and maladaptive effects on neuroplasticity, which is essential for processes such as memory, learning, and cognition [35,40,41] and also plays a role in mood disorders [42].

The impact of chronic versus acute stress on neuroplasticity differs. Chronic stress is often associated with maladaptive changes and pathologies such as depression, anxiety, and cognitive impairments. This is partly due to allostatic load, the cumulative burden of chronic stress, and life events [43]. Stress-induced neuronal plasticity disruptions, including AMPAR dynamics alterations, are well-documented [36]. Given the connection between neuronal plasticity and tonotopic organization in auditory pathways, such changes have implications for auditory disorders like tinnitus.

**Figure 3:**
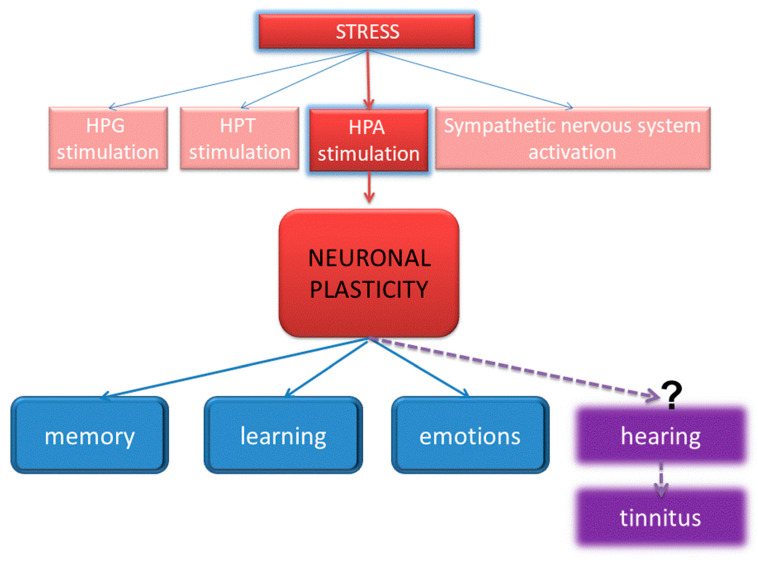
Hypothetical model of chronic stress effects on neuroplasticity through the HPA-axis. The known connections between the HPA-axis stimulation and neuronal plasticity affecting memory, learning, and emotions are shown in solid arrows. The connection with the plastic changes affecting hearing and resulting, e.g., in tinnitus, is still purely hypothetical; thus, it is shown with dashed arrows. HPG, the hypothalamic-pituitary-gonadal axis; HPT, the hypothalamic–pituitary–thyroid axis; HPA, the hypothalamic–pituitary–adrenal axis. Reproduced from Mazurek et al. [17] under Creative Commons Attribution License.

Figure 3 illustrates a theoretical relationship between chronic stress and neuroplasticity and proposes how stress may lead to changes in neural pathways, which could potentially contribute to the development or exacerbation of tinnitus.

### 1.4. Corticosteroids and AMPA Receptors

Corticosteroids exert their effects through glucocorticoid receptors (GRs) and mineralocorticoid receptors (MRs). These receptors operate at both the membrane level and within the nucleus, enabling corticosteroids to rapidly influence cellular processes and induce long-term changes in gene expression [44]. GRs are thought to enhance the expression of AMPA receptors (AMPAR) involved in post-synaptic neuroplasticity, while MRs affect the trafficking of AMPAR [17,37].

GRs are found throughout the central nervous system (CNS), whereas MRs are primarily located in limbic regions, including the hippocampus, lateral septum, amygdala [45,46,47], and prefrontal cortex [48]. The presence of both MR and GR receptors has been confirmed in the inner ears of rodents, where they display distinct expression patterns. MRs are present in various structures, such as the organ of Corti, stria vascularis, spiral ligament, spiral ganglion cells, and the cochlear nerve [32,33,34]. However, the expression patterns of MR and GR in auditory regions beyond the inner ear have yet to be investigated.

Stress increases the release of glutamate in various brain regions [49]. In the mouse hippocampus, this process occurs rapidly through membrane-bound MRs, resulting in an increase in AMPA-mediated excitatory postsynaptic currents [50]. Similar mechanisms in the inner ear may contribute to tinnitus by stimulating the auditory nerve or causing local glutamate excitotoxicity [51,52]. In the stria vascularis, MRs may play a significant role in regulating ionic and water balance, which is essential for proper auditory function, as the ionic gradient is necessary for efficiently transducing auditory signals [1].

### 1.5. Exploring the Impact of Corticosterone on AMPARs

The effects of corticosteroids on AMPA receptors have been extensively studied in cognitive sciences, focusing on morphological and molecular changes in the central nervous system. However, their role in the auditory system has received less attention. This is notable given the established function of AMPARs in fast excitatory signaling throughout the auditory pathway, from the initial synapse to the auditory cortex, as well as their involvement in neuronal plasticity, which may contribute to the adaptations in tonotopic organization observed in tinnitus.

Tinnitus affects an estimated 14% of the global adult population—over 740 million people—and can be induced or exacerbated by various pathomechanisms, including emotional stress. Understanding how stress impacts the auditory system and potentially leads to disorders such as tinnitus, sudden hearing loss, or Meniere’s disease could be essential for developing effective therapies.

This scoping review aims to gather existing bibliographical information regarding the effects of corticosterone on the levels and localization of AMPARs. Our goal is to determine whether there is a consistent effect across the rodent’s nervous system. The primary focus is to identify research explicitly addressing the impact of corticosterone on AMPARs in the auditory system. Additionally, the secondary objective is to find any consensus regarding the effects of corticosterone on AMPARs throughout the rodent nervous system.

## 2. Materials and Methods

### 2.1. Search Strategy and Databases

This scoping review adhered to the guidelines in Preferred Reporting Items for Systematic Review and Meta-Analyses (PRISMA) [53]. Figure 3 shows the PRISMA flowchart that illustrates the study selection process. This scoping review protocol was developed and registered on the Open Science Framework (OSF) to enhance transparency and replicability. The protocol, including our detailed search strategy, eligibility criteria, and data extraction approach, is available at https://osf.io/wzg7n (accessed on 23 October 2024) registration DOI: https://doi.org/10.17605/OSF.IO/WZG7N (accessed on 23 October 2024).

Using EndNote 21, a reviewer conducted searches in the electronic databases PubMed, Web of Science. A search was also carried out in OVID EMBASE and accessed online. The number of databases utilized in this study adhered to the recent guidelines for scoping reviews [54]. The searches were performed on 1 August 2023, with no date restrictions. Two searches, A and B, with two different combinations of MeSH terms, were performed:

- Search A: (AMPA) AND (corticosterone)
- Search B: (glutamate receptor) AND (corticosterone)

A total of 536 publications were identified through database searches and reference lists. After the duplicates were removed, 288 articles remained. These were imported to the web-based citation management system Rayyan [55] for further screening.

### 2.2. Screening Process and Inclusion and Exclusion Criteria

Two independent reviewers screened the titles and abstracts of the 288 articles.

The inclusion criteria were as follows:

- Publication language—English;
- Type of publication—original research;
- Focused on one or more of the following molecules: AMPA, AMPA1, AMPA2, AMPA3, AMPA4, GluR1, GluR2, GluR3, GluR4, GluA1, GluA2, GluA3, GluA4, GriA1, GriA2, Gria3, Gria4.

The exclusion criteria were as follows:

- Publication language other than English;
- Type of publication—review, letter to editor, editorial;
- Focused on NMDA or mGlur.

This review focuses on AMPARs because of their well-established role in neuronal plasticity and their crucial function in fast synaptic transmission, both of which are essential for auditory processing. In contrast to NMDA and mGluR receptors, which have broader modulatory functions, AMPARs are specifically responsible for mediating rapid excitatory signaling.

After excluding off-topic articles, those with the wrong publication type, or studies not conducted in English, 33 articles remained for full-text screening.

### 2.3. Study Eligibility and Data Extraction

**Figure 4:**
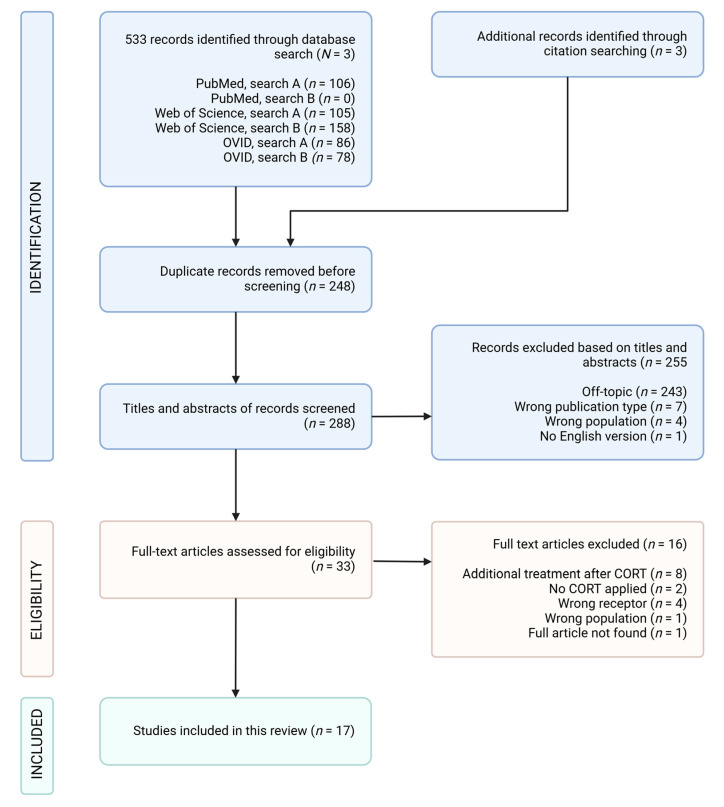
PRISMA flow diagram of study selection for scoping review. Abbreviations: “N” signifies the number of databases; “n” signifies the number of publications; CORT, corticosterone. Created with Biorender.com (accessed on 21 January 2025).

Out of the initial 33 articles, 17 remained after the full-text screening. The exclusions occurred for several reasons: some studies involved additional treatments (such as adrenalectomy, exposure to stress, or prolactin treatment) following CORT exposure, while others had no CORT exposure. Additionally, some articles referenced the incorrect receptor or population, and one full-text article was inaccessible. The screening process is illustrated in a PRISMA flow diagram in Figure 4.

## 3. Results

All extracted data are provided in Supplementary Table S1, S1_Extracted_Data.

### 3.1. Characteristics of Included Studies

Out of the seventeen included studies, fourteen primarily aimed to investigate the impact of corticosterone on AMPA receptor trafficking and regulation, while the remaining three focused on the effects of stress on memory consolidation, cognitive function, and behavioral outcomes. The studies were published between 2008 and 2022 by 23 research groups from Canada, China, France, the Netherlands, Poland, Spain, the UK, and the USA.

### 3.2. Methodology of Included Studies

#### 3.2.1. Experimental Models and Areas

The 17 included studies included 39 experiments in total. The studies used various rodent models: wild-type C57BL/6N mice (1 study), ICR mice (2 studies), Wistar rats (3 studies), and Sprague-Dawley rats (8 studies). The specific rat strain was not specified in four studies that used primary neuronal cell cultures, with authors referring to previous publications instead.

The experiments in the studies can be divided as follows:

- CORT application on isolated tissues (*n* = 2)
- CORT application on dispersed tissues or cell lines (*n* = 15)
- CORT application on live animals (*n* = 22)

All studies investigated AMPARs in central nervous system (CNS) tissues, but none in the peripheral nervous system (PNS).

The majority of experiments (*n* = 26) focused on the hippocampus, with fewer studies examining other CNS regions—the prefrontal cortex (*n* = 3), amygdala (*n* = 3), primary motor cortex (*n* = 4), sensorimotor cortex (*n* = 4), and visual cortex (*n* = 1).

#### 3.2.2. CORT Administration and Exposure

CORT was administered to live animals through various routes: intraperitoneal injection (*n* = 3), subcutaneous injection (*n* = 1), pellet implantation (*n* = 1), and drinking water (*n* = 1).

In vitro experiments were conducted by applying CORT to neuronal cell cultures (*n* = 15) or brain tissue slices/punches (*n* = 2).

CORT exposure durations of the 39 included experiments were categorized by us as acute (<1 h, *n* = 14), subchronic (180 min, *n* = 6), and chronic (>24 h, *n* = 19).

Acute exposure (*n* = 14) typically involved concentrations around 100 nM for 10 to 30 min. Chronic models (*n* = 19) used concentrations ranging from 5–18 mg/kg body weight over 6 h to 35 days. The subchronic models (*n* = 6) used 100 nM CORT for 180 min.

The time elapsed between CORT exposure and measurement of AMPA subunit levels varied across experiments. For acute exposures (<1 h), measurements were taken between 1 min and 240 min post-exposure, but some were extended overnight. Subchronic exposure experiments (180 min) typically measured results from 60 min to overnight after CORT exposure. Chronic exposure studies (≥24 h) involved longer intervals, ranging from immediate measurement to several days, depending on experimental protocols and tissue collection schedules. The two experiments did not specify the time interval between CORT exposure and measurement in their methodologies.

#### 3.2.3. Techniques Used in Included Experiments

Out of the 39 experiments conducted in the studies, 22 utilized Western blotting to measure the levels of membrane AMPAR subunit proteins. Twelve experiments used immunolabeling to visualize synaptic and/or total surface AMPAR subunits. Of these, 8 used immunocytochemistry, and 4 used immunofluorescence. Three experiments used fluorescence recovery after photobleaching (FRAP), and two utilized single-particle tracking to visualize AMPAR subunit mobility or surface AMPAR subunit trafficking. Most experiments used antibodies specific for GluR1 (*n* = 19) and/or GluR2 (*n* = 12). One used antibodies against GluR2/3, two GluR3, and three GluR4.

### 3.3. Outcomes of Included Studies

#### 3.3.1. Summary of Findings

The 17 studies included a total of 39 experiments. The findings are detailed in the following sections and summarized in the corresponding tables and figures. Table 1 summarizes regional outcomes, reporting increases, decreases, or no changes in AMPAR protein levels and localization. More details for live animal and neuronal culture experimental models are presented in Table 2 and Table 3, respectively, and are described in Section 3.3.2 and Section 3.3.3. Isolated tissue experimental models are described in Section 3.3.4. The data in these sections can also be viewed collectively in Supplementary Table S2, S2_Experimental_Details_and_Outcomes.

#### 3.3.2. Effects of CORT Exposure on AMPAR Levels in Live Animal Models

Out of 39 experiments, 22 investigated the effects of CORT on AMPAR levels in live animal models. The studies primarily used male Sprague-Dawley and Wistar rats, with one study using male ICR mice and one using male and female C57BL/6N mice. The animals ranged in age from 3 to 12 weeks. In cases where only weight was provided without age information, we estimated the age using a weight chart from Charles River Laboratories [56]. CORT was administered through drinking water, subcutaneous or intraperitoneal injection, and had both acute and chronic exposure durations. In the following presentation of outcomes, the terminology from the original published interpretations of results is used where applicable.

Effects of Acute Exposure (<1 h)

Acute exposure to CORT produced varying effects in different animal models. In Wistar rats, a single subcutaneous injection of CORT did not affect the levels of GluR1 or GluR2 in the primary motor cortex [57]. In contrast, a single intraperitoneal injection of CORT in ICR mice led to a decrease in the levels of GluR1 and GluR2 in the hippocampus [58]. Furthermore, intraperitoneal injection of CORT increased the mobility of GluR1 in the visual cortex of C57BL/6N mice [59].

Effects of Subchronic Exposure (3 h)

No experiments involving live animal models were found within this duration of exposure.

Effects of Chronic Exposure (>24 h)

In male Sprague-Dawley rats, chronic exposure to CORT in drinking water for 14 days resulted in decreased levels of GluR1 in the hippocampus, while levels increased in the amygdala [60]. After the same exposure followed by a 14-day washout period, there was no significant change in GluR1 levels in the hippocampus, but levels continued to rise in the amygdala [60]. A study found that 21 days of daily intraperitoneal injections of CORT increased the levels of GluR1 and GluR2 in the hippocampus of 3-week-old rats [61]. However, in 8-week-old rats, this same treatment did not affect the levels of GluR1, GluR2, GluR3, or GluR4 in the hippocampus [61]).

**Figure 5:**
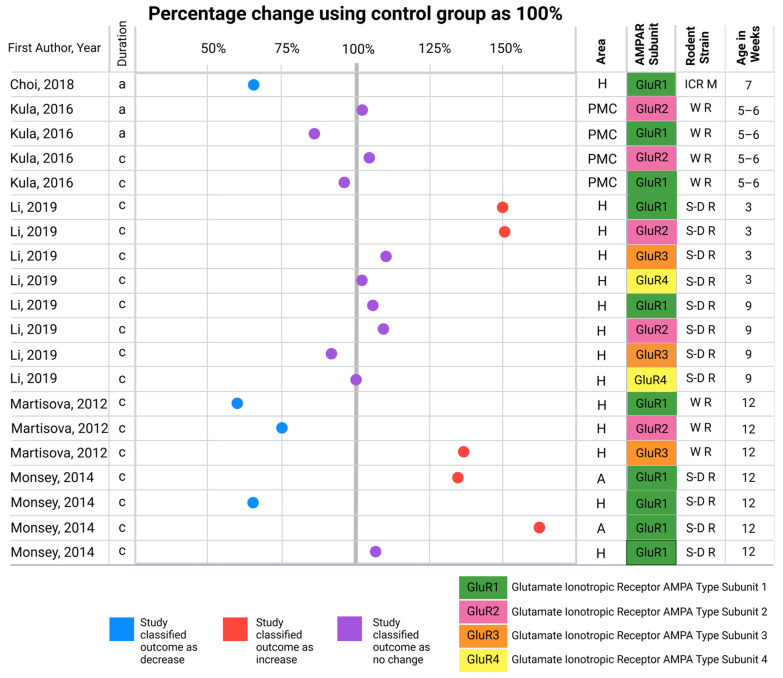
Comparative summary of findings from included studies on changes in AMPAR subunit protein levels in the hippocampus, primary motor cortex, and amygdala following acute (<1 h) or chronic (>6 h) corticosterone (CORT) exposure. The figure summarizes data reported in included studies, where AMPAR subunit protein levels or localization (surface or synaptic) were assessed using Western blot analyses. Results are plotted as categorized by the studies into increases, decreases, or no changes relative to untreated controls (control group set at 100%). The figure includes information on the AMPAR subunit, rodent strain, and the age of the animals in weeks. The respective references [57,58,60,61,62] are sorted in alphabetical order. This figure includes the results, as reported in the studies, without considering the measurement time after CORT exposure. Abbreviations: a, acute; c, chronic; H, hippocampus; PMC, primary motor cortex; A, amygdala; ICR M, ICR mouse; W R, Wistar rat; S-D R, Sprague-Dawley rat. Created with Biorender.com (accessed on 21 January 2025).

In Wistar rats, chronic CORT exposure over 35 days through subcutaneously implanted pellets reduced the levels of GluR1 and GluR2/3 in the hippocampus [62]. Conversely, the level of GluR4 increased. Additionally, after 7 days of receiving two subcutaneous injections per day, there was no change in the levels of GluR1 or GluR2 in the primary motor cortex of Wistar rats [57] (Table 2, Figure 5).

**Table 2:** Experimental details and observed results of corticosterone exposure on AMPARs in live animals. The respective references are sorted in alphabetical order.

First Author and Year	Animal	Age in Weeks	Sample Size	Area	CORT Exposure Duration	CORT Concentration	Time from CORT Exposure to Reading	Measuring Method	Main Extracted Findings
Chen, 2021 [59]	C57BL/6N mice, M + F	8–16	5	Visual cortex	One intraperitoneal injection	5 mg/kg	60, 120, and 180 min post-injection	FRAP	SEP-GluA1 fluorescence recovery significantly increased to 90% three hours after injection of CORT, suggesting that CORT injection causes a shift from a nearly equal split between mobile and immobile pools to an almost entirely mobile pool of GluA1-containing receptors within cortical synapses.
Choi, 2018 [58]	ICR mice, M	7	5 for WB, 5 for IC	Hippocampus	One intraperitoneal injection	10 mg/kg	Not specified	WB + IC	Exposure to CORT showed reduced trafficking of AMPAR 1/2 into the synapse due to microtubule destabilization.
Kula, 2016 [57]	Wistar rats, M	5–6	6–8	Primary motor cortex	One subcutaneous injection	10 mg/kg	Tissue collection 2, 4, and 7 days after the last CORT administration	WB	CORT did not influence the protein levels of GluA1 and GluA2 subunits.
Li, 2019 [61]	Sprague-Dawley rats, M	3	5–6	Hippocampus	21 days of 1 intraperitoneal injection/day	5 mg/kg	Tissue collection 48 h or 4 weeks after last injection	WB	In adolescents, both the GluA1 and GluA2 subunits were significantly upregulated by CORT treatment. GluA3 or GluA4 expression levels remained unchanged.
Li, 2019 [61]	Sprague-Dawley rats, M	9	5–6	Hippocampus	21 days of 1 Intraperitoneal injection/day	5 mg/kg	Tissue collection 48 h or four weeks after the last injection	WB	No significant changes were observed in the GluR1, 2, 3, or 4 AMPA receptor subunits following CORT treatment in adults.
Martisova, 2012 [62]	Wistar rats, M	12	6	Hippocampus	35 days of subcutaneously implanted pellets	18 mg/kg	Tissue collection 35 days after pellet implantation	WB	GluR1 and GluR2/3 expression were decreased in chronic treatment with CORT. GluR4 increased.
Monsey, 2014 [60]	Sprague-Dawley rats, M	12	9	Amygdala	14 days in drinking water (+subset with 14-day washout period)	50 mg/mL drinking water	Experiment 1: tissue collection 0 days after the last CORT administration. Experiment 2: tissue collection 14 days after the last CORT administration	WB	Chronic CORT exposure resulted in an increase in GluR1 protein expression in the lateral amygdala. The enhanced expression of GluR1 persisted following the 14-day recovery period.
Monsey, 2014 [60]	Sprague-Dawley rats, M	12	9	Hippocampus	14 days in drinking water (+subset with 14-day washout period)	50 mg/mL drinking water	Experiment 1: tissue collection 0 days after the last CORT administration. Experiment 2: tissue collection 14 days after the last CORT administration	WB	Chronic CORT exposure resulted in a decrease in GluR1 protein expression in the hippocampal area CA3. The enhanced expression did not persist following the 14-day recovery period.

Abbreviations: M, male; F, female; CORT, corticosterone; WB, Western blot; IF, immunofluorescence; IC, immunocytochemistry; FRAP, fluorescence recovery after photobleaching.

#### 3.3.3. Effects of Exposure to CORT on AMPAR Levels in Neuronal Cell Cultures

Fifteen out of the thirty-nine experiments investigated the effects of acute, subchronic, and chronic exposure to CORT on AMPAR levels in neuronal cell cultures. The following presentation of results uses terminology from the original published interpretations where applicable.

Effects of Acute Exposure (<1 h)

Acute exposure (10–30 min) to 100 nM CORT in 6 out of 8 experiments resulted in increased surface content or cluster density of GluR1 and GluR2. Specifically, studies observed elevated GluR1 in prefrontal cortex cultures [63,64] and increased GluR2 levels in hippocampal cultures [65,66,67]. Additionally, Groc et al. (2008) reported increased GluR2 mobility in hippocampal cultures [65]. However, one study using a lower concentration (30 nM) of CORT for 15 min showed no significant changes in GluR1 or GluR2 surface expression in hippocampal cell cultures [68].

Effects of Subchronic Exposure (3 h)

Experiments investigating subchronic exposure (180 min) to 100 nM CORT resulted in upregulation of GluR1 and GluR2 surface expression in hippocampal cultures (Martin et al., 2009 [69]). Subchronic exposure also led to an increase in the mobile fraction of GluR2 containing AMPARs (Martin et al., 2009 [69], Xiong et al., 2015 [70]).

Effects of Chronic Exposure (>24 h)

Chronic exposure to CORT (ranging from 24 h to 7 days) demonstrated varied effects. Seven days of exposure to 100 nM CORT decreased total GluR1 cluster density in prefrontal cortex cell cultures (Yuen et al., 2012 [71]), while a 24 h exposure to 50 mM CORT enhanced GluR1 fluorescence intensity in amygdala cell cultures (Li et al., 2021 [61]).

These findings are summarized in Table 3.

**Table 3:** Experimental details and observed outcomes of corticosterone exposure on AMPARs in neuronal cell cultures. The respective references are sorted in alphabetical order.

First Author and Year	Animal	Area	Sample Size	CORT Exposure Duration	CORT Concentration	Time from CORT Exposure to Reading	Measuring Method	Main Extracted Findings
Groc, 2008 [65]	Sprague-Dawley rats	Hippocampus	5 hippocampal cultures	20 min	100 nM	1–150 min	IF	CORT triggers time-dependent increases in GluR2-AMPAR surface mobility and synaptic surface GluR2 content. The peak stimulatory effects of CORT on surface GluR2-AMPAR mobility and relative synaptic content were observed at 150 min after application.
Li, 2021 [72]	Sprague–Dawley rats	Amygdala	4 wells	24 h	50 mM	Overnight **	IC	CORT significantly enhanced the surface fluorescence intensity, cluster density and cluster size of GluA1-positive neurons
Liu, 2010 [63]	Not stated (rat) *	Prefrontal cortex	24 neurons	30 min	100 nM	Experiment 1: 90–240 min. Experiment 2: 120 min	IC	CORT profoundly increased surface GluR1 cluster density.
Martin, 2009 [69]	Not stated (rat) *	Hippocampus	IC: not specified; FRAP: 10 spines	180 min	100 nM	60–180 min	IC, FRAP	Both GluR1 and GluR2 surface expression are increased by corticosterone, but GluR2 is more sensitive and increases to a greater extent than GluR1. No change in GluR2 after 1 h of CORT but pronounced effects after 3 h. CORT mobilizes usually synaptically anchored surface-expressed AMPARs.
Sarabdjitsingh, 2014 [66]	Sprague-Dawley rats	Hippocampus	19 neuronal fields analyzed	10 min	100 nM	60–120 min	SPT	A single CORT pulse significantly increased the surface diffusion of GluA2–AMPAR. The introduction of a second pulse of CORT 60 min after the first abolished this effect.
Sarabdjitsingh, 2016 [67]	Sprague-Dawley rats	Hippocampus	Sample size not reported; number of experiments = 7	10 min	100 nM	5–240 min	SPT	GluA2-AMPAR surface trafficking in hippocampal neurons is particularly responsive to the first pulse of CORT, less consistently to the 2nd and 3rd pulse, and insensitive to the 4th pulse.
Xiong, 2015 [70]	Wistar rats	Hippocampus	surface quantification > 10 cells; FRAP: 10–16 cells	180 min	100 nM	Overnight **	IC, FRAP	CORT increased the surface expression of GluA1 and GluA2 AMPAR subunits. CORT increased the mobile fraction of GluA2-containing AMPAR.
Yuen, 2011 [64]	Sprague-Dawley rats	Prefrontal cortex	12 neurons	20 min	100 nM	60–240 min	IC	CORT treatment induced a significant increase in synaptic GluR1 cluster density.
Yuen, 2012 [71]	Not stated * (rat)	Prefrontal cortex	12 neurons	7 days	100 nM	Not specified	IC	Chronic CORT treatment reduced the cluster density of total GluR1 and synaptic GluR1.
Zhou, 2012 [68]	Not stated * (rat)	Hippocampus	12–20 neurons	15 min	30 nM	Overnight **	IF	CORT did not change GluA1 and GluA2 surface expression.

* The publication included in this review referred the reader to previous publications. ** As stated in the paper. Abbreviations: CORT, corticosterone; WB: Western blot; IF, immunofluorescence; IC, immunocytochemistry; FRAP, fluorescence recovery after photobleaching; SPT, single particle tracking.

#### 3.3.4. Effects of CORT Exposure on AMPAR Levels in Isolated Tissue Samples

Two of the thirty-nine experiments described in one study examined the effects of CORT on AMPAR levels in isolated tissue samples from male Sprague-Dawley rats aged 7 to 10 weeks. Both exposures were acute. Where applicable, terminology from the original published interpretations of the results is used in the following presentation of outcomes.

Effects of Acute Exposure (<1 h)

Acute exposure to 200 nM CORT for 30 min in Sprague-Dawley rats induced no change in the surface expression of GluR1 or GluR2 in the sensorimotor cortex [73].

Effects of Subchronic Exposure (3 h)

No experiments in isolated tissue models were identified within this exposure duration category.

Effects of Chronic Exposure (>24 h)

No experiments in isolated tissue models were identified within this exposure duration category.

## 4. Discussion

### 4.1. Overview of Findings

This scoping review aimed to evaluate the effects of CORT exposure on AMPAR levels and localization in the rodent nervous system. It included 17 studies, totaling 39 experiments, published between 2008 and 2022 by 23 research groups from Canada, China, France, the Netherlands, Poland, Spain, the UK, and the USA. To assess outcomes, the experiments used Western blotting, immunolabeling, or single-particle tracking. Most studies focused on GluR1 and GluR2, with limited use of GluR3 and GluR4. Most studies involved the hippocampus, and none investigated AMPAR dynamics in the auditory or peripheral nervous systems.

To facilitate comparisons, we categorized the experiments based on the duration of corticosterone exposure as acute, subchronic, and chronic. Additionally, we identified three types of experimental models used across the studies: primary cell cultures, live animals, and isolated tissues. The findings indicate inconsistencies across these models and regions, making it challenging to draw clear conclusions about corticosterone’s effects on AMPAR dynamics.

In neuronal cell cultures, exposure to CORT generally resulted in increased surface localization and mobility of AMPARs during acute and subchronic exposure conditions. However, limited data from one isolated tissue study showed that acute exposure did not affect the synaptic content of GluR1 or GluR2 in the sensorimotor cortex.

The results in live animal models were inconsistent. The protein levels of AMPAR subunits after CORT exposure—most of which were chronic—varied significantly, with some studies reporting increases, others reporting decreases, and some showing no change. These variations appeared to be independent of the central nervous system region. Given the differences among these models, we will discuss each category individually below.

### 4.2. Live Animal Models Show High Variability and Need for Stringent Protocols

Synthesizing results from live animal models proved challenging due to variability in outcomes, which could be attributed to various known and lesser-known factors. These factors include differences in the age, species, and strains of the rodents used across different studies. For example, some studies utilized young Sprague-Dawley rats, whereas others employed older ICR mice, which may respond differently to CORT due to developmental or genetic differences. Additionally, the timing between CORT exposure and AMPAR measurement varied significantly, with one study omitting this information entirely. Furthermore, the current study did not account for the chronic cumulative effects of CORT exposure throughout the animals’ bodies, which likely changed over time and could have influenced the final results.

CORT administration methods—whether through drinking water, subcutaneous injections, or intraperitoneal injections—likely contributed to variability in results by affecting bioavailability and metabolism in different ways. Additionally, repeated injections may serve as stressors. Pérez-Valenzuela et al. [74] have noted that repeated vehicle injections can impact auditory attention in control animals, indicating that the injection process itself introduces stress. Bagley and Moghaddam [75] have observed that repeated stress, such as tail-pinch procedures, affects glutamate efflux dynamics in the prefrontal cortex and hippocampus over time. Initially, there is an increase in glutamate response, followed by a reduced response to subsequent exposures. These findings suggest that the stress induced by handling or injections may influence neurochemical processes like glutamate efflux, potentially affecting AMPAR regulation and the experimental outcomes of the studies included in this scoping review.

Unreported or less-controlled factors may have also contributed to the variability observed across the studies. These factors include differences in housing conditions, such as cage size, population size, environmental enrichment, light–dark cycles, temperature, humidity, ventilation, and noise exposure. Additionally, variations in animal handling practices, such as the frequency, method, and timing of handling in relation to the nocturnal activity patterns of rodents, acclimatization duration, and diet, may also play a role. While some studies document many of these factors, others do not provide this information.

Species-specific stress responses and the predominance of male subjects may contribute to variability in outcomes. None of the experiments compare animals of different sexes despite known differences in human stress responses based on biological sex [76].

Most studies have focused on chronic exposure, complicating comparisons with neuronal cell cultures and isolated tissue models. Additionally, the predominance of Western blotting for measuring AMPAR levels in 19 of the 22 experiments, compared to other methods like immunolabeling or single-particle tracking, further constrains cross-model comparisons.

The variability in methodologies and outcomes, along with the inherent complexity of studying stress-related effects in stressed animals, underscores the necessity for standardized experimental protocols and improving reporting practices.

### 4.3. Limited Isolated Tissue Models and Region-Specific Changes

In contrast to the variability found in live animal models, studies using isolated tissues can provide greater experimental control. However, these models are significantly underrepresented, with only one study included in this research. Thacker et al. [73] reported that acute exposure to 200 nM CORT for 30 min did not significantly affect the synaptic levels of GluR1 or GluR2 in the sensorimotor cortex. However, an increase in the total amount of phosphorylated GluR1 was observed. The phosphorylation of the GluR1 subunit is believed to play a crucial role in regulating the synaptic presence of AMPARs by reducing the internalization of these receptors, thereby increasing their availability at the synapse [77]. As noted in the introduction (Section 1.2), the trafficking of AMPAR subunits remains a complex and evolving area of study.

Thacker et al. proposed that the observed phosphorylation may indicate that mineralocorticoid receptor activation primes AMPARs for synaptic insertion. They referenced studies showed that low doses of corticosterone (less than 200 nM) activate the MR, leading to increased GluR1 insertion [69,78,79]. Additionally, they suggested that this priming process might require other factors, such as activity-dependent stimulation or prolonged receptor priming. This assertion is supported by findings demonstrating that exposure to corticosterone enhances GluR1 phosphorylation and surface protein levels [65,80].

One study by Whitehead et al. [80] utilized an isolated tissue model but was excluded from this review because their corticosterone experiments fell outside our predefined scope. Nevertheless, they reported increased GluR1 surface localization in hippocampal slices treated with dexamethasone, a synthetic corticosteroid commonly used in clinical practice. Although dexamethasone was not the primary focus of this review, its effects underscore the potential of corticosteroids to influence AMPAR dynamics. Further investigation into the impact of dexamethasone on AMPARs could provide valuable insights into how commonly used corticosteroid treatments affect synaptic plasticity and neurotransmission.

Based on the single study included, we cannot determine whether the observed differences in outcomes related to acute CORT exposure in isolated tissues and neuronal cultures are due to regional variations. For instance, there may be a lack of synaptic changes in the sensorimotor cortex compared to changes in the hippocampus and prefrontal cortex. Additionally, factors such as MR activation, stimulation-dependent insertion into the synapse, prolonged receptor priming, or the specific experimental model used could also play a role.

Future studies examining the effects of CORT in isolated tissues from various regions of the nervous system could provide valuable insights into regulating AMPAR subunits under controlled conditions. Furthermore, neuronal cell cultures can offer a carefully regulated environment to explore these mechanisms.

### 4.4. Concentration and Timing-Dependent in AMPAR Level Increases in Neuronal Cell Cultures

The consistency in neuronal cell culture models contrasts with the variability in live animal studies and the limited data available from isolated tissues. Acute and subchronic exposure to 100 nM CORT generally increased the protein levels and mobility of GluR1 and GluR2 in neurons located in the hippocampus and prefrontal cortex. Some studies have indicated time-dependent effects, with the most significant changes occurring between 60 and 150 min after exposure [65,66]. While these increases in receptor localization or surface levels do not necessarily suggest increased AMPAR presence at synapses, they may indicate enhanced lateral diffusion near the synapse, thereby contributing to a dynamic reserve of receptors. However, Martin et al. [69] observed that after 3 h of exposure to 100 nM CORT, there was an increase in the amplitude of miniature excitatory postsynaptic currents (mEPSCs). This suggests that receptor mobility or surface-level alterations can enhance synaptic functionality.

Zhou et al. [68] found that 30 nM CORT applied for 15 min did not significantly change the surface levels of GluR1 or GluR2 in hippocampal cultures. This suggests that CORT concentration may play a role in the degree of modulation of AMPA receptors. In contrast, Martin et al. [69] reported significant increases in the lateral mobility of GluR2 at the same concentration after 3 h, highlighting the importance of the duration of exposure.

**Figure 6:**
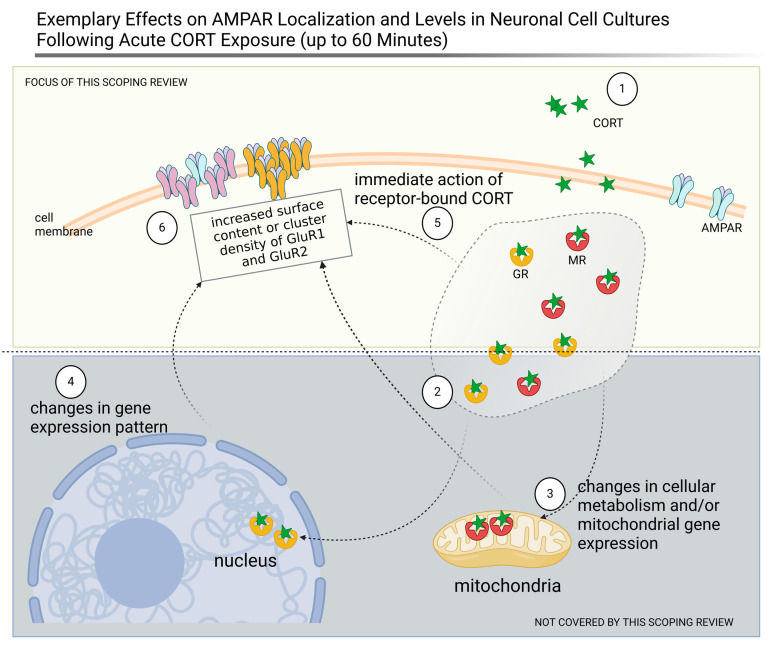
Short-term exposure to 100 nM corticosterone (CORT) enhances the levels and localization of AMPAR in rodent neuronal cultures. The addition of 100 µM CORT represented by green stars (1) induces its binding (2) by the glucocorticoid (GR) and mineralocorticoid (MR) receptors; the receptors translocated to the mitochondria, where they influence the metabolism and mitochondrial gene expression (3), and/or to the nucleus, where they regulate the gene expression pattern (4). Moreover, when they bind their ligand, membrane-bound GRs and MRs (5) can enhance the mobility of AMPAR directly, while the overall AMPAR levels are regulated through transcription and translation (6). Based on [44,69]. Created with Biorender.com (accessed on 21 January 2025).

The consistent findings in neuronal cell cultures indicate that short-term CORT exposure at specific concentrations has similar effects on AMPAR dynamics in hippocampal and prefrontal cortex neurons, which is consistent with the role of these brain regions in stress-related neuroplasticity (Figure 6).

The results from studies on chronic exposure to CORT showed more variability. Yuen et al. [71] found that a 7-day exposure to 100 nM CORT reduced AMPAR density in the prefrontal cortex. In contrast, Li et al. [72] reported an increase in AMPAR levels in the amygdala after a shorter 24 h exposure at a much higher concentration of 50 µM. These differing results are difficult to generalize since they are based on only two studies, but they may suggest region-specific responses to prolonged CORT exposure. This observation aligns with existing literature, which indicates that chronic stress can reduce dendritic spines in the prefrontal cortex [81], while it may induce dendritic hypertrophy and increase spine density in the amygdala [82,83].

### 4.5. Cross-Model Patterns and Research Gaps

Our scoping review examined the consensus on the effects of CORT exposure on AMPAR levels and localization in the rodent nervous system. Inconsistent findings across various experimental models and brain regions complicate this determination. While some patterns have emerged, significant variability highlights gaps in the research.

Neuronal cultures indicate that both acute and subchronic CORT exposure increases protein levels and mobility in the hippocampus and prefrontal cortex. However, since these studies focused on only two regions, we cannot conclude a universal effect throughout the nervous system. The consistent impact seen in multiple studies suggests repeatability, yet more research in other areas is essential.

Research on isolated tissue models is limited, with only one study in this review on acute CORT effects on AMPARs. This limits our understanding of CORT’s direct impact on AMPAR dynamics in ex vivo environments. One study reported increased AMPAR protein levels in the amygdala with chronic exposure, while another found decreased levels in the prefrontal cortex, consistent with existing literature on stress responses [81,82,83]. However, the variability in exposure durations and concentrations among these studies makes generalization difficult.

The highly variable results from live animal studies further complicate comparisons and raise questions about the long-term effects of CORT exposure, the regulatory mechanisms involved, and how organisms adapt to chronic versus acute stress. Additionally, variability in stress responses among species, strains, and sexes contributes to these inconsistencies. The oversight of these factors reveals a significant research gap.

The timing of CORT exposure is significant for the AMPAR response, as demonstrated by Groc et al. and Sarabdjitsingh et al. [50,51]. This poses challenges in analyzing results from studies where exposure-to-measurement intervals were not reported.

A study by Kula [57] emphasized the GluR1 and GluR2 subunits, as over 80% of native AMPA receptors are GluR1R2 heteromers. This focus explains the limited research on GluR3 and GluR4. While GluR1 and GluR2 have received the most attention—partly due to research interest and available reagents—this narrows our understanding of how corticosterone affects all of the AMPAR subunits. Recent research by Rutherford et al. [84] highlights the role of GluR3 subunits in assembling AMPARs with GluR2 and GluR4 at cochlear synapses, indicating the need for more studies on these less-explored subunits and regions.

### 4.6. Implications for the Auditory System

Given the essential role of AMPA receptors in auditory processing, it is crucial to investigate whether exposure to corticosterone may lead to changes in their localization and mobility, as this would affect auditory function. While acute and subchronic exposure to CORT consistently impacts neuronal cell cultures from various regions, studies using different models show variability depending on the region and experimental conditions (see Table 1). This suggests that other factors, such as experimental design, duration, and concentration or type of exposure, play a significant role. Similar to how different regions of the central nervous system (CNS) respond in complex and varied ways to CORT exposure at different concentrations and durations, we may observe comparable responses in peripheral areas, including the inner ear.

Disruptions in tonotopic organization, which rely on neuronal plasticity, have been linked to auditory disorders such as tinnitus. AMPARs are crucial for mediating synaptic changes, play an essential role in maintaining neuronal plasticity, and may help sustain the stability of tonotopic organization. Exploring if and how CORT affects AMPAR dynamics in the auditory system could provide valuable insights into stress-related changes in tonotopy and their potential impact on auditory dysfunction.

One crucial mechanism through which CORT exerts its effects involves its action on two types of receptors: mineralocorticoid receptors (MRs) and glucocorticoid receptors (GRs). MRs influence the trafficking of AMPA receptors [17,37]. Unlike the widely expressed GRs, MRs are unevenly distributed throughout the nervous system, which may account for regional differences in AMPAR responses to CORT. This review has identified changes in AMPAR expression in brain regions where MRs are abundant [45,48]. Similar alterations may occur in the auditory periphery, where MRs are present in the organ of Corti, stria vascularis, and spiral ganglion cells [32,33,34].

The corticosteroid receptor balance hypothesis [85] suggests that chronic stress can disrupt the balance between MRs and GRs, leading to overactive MRs and hypocortisolism. This imbalance may exacerbate stress-related conditions such as tinnitus. Hypocortisolism has also been observed in tinnitus patients experiencing psychosocial stress [7]. An imbalance in the MR/GR could contribute to what is described as the “cortisol-sensitive tinnitus connectome,” although further studies are needed to confirm this connection [51,86]. The MR/GR balance may influence AMPAR dynamics. At lower concentrations, CORT acting through MRs enhances synaptic excitability; at higher concentrations, GRs affect AMPAR expression and trafficking [78]. This receptor-dependent mechanism may contribute to changes in AMPAR dynamics and support region-specific responses. Investigating MR expression patterns in the auditory pathway may help clarify how MR/GR dynamics impact AMPAR function and sensory processing, particularly in relation to stress-related auditory conditions. However, no studies have yet explored the influence of CORT on AMPARs in the auditory system.

### 4.7. Limitations

This review is limited by variability in experimental protocols across the included studies, affecting the consistency of findings. Most research focused on the hippocampus and prefrontal cortex, with little data on other brain regions or peripheral areas, limiting the generalizability of results.

Another drawback is the ambiguity in data reporting and internal categorizations. For example, distinguishing between surface, membrane, and synaptic protein levels can be challenging. In Table 1, Table 2 and Table 3, we simplified changes and extracted outcomes directly from the studies, but the level of detail depended on the authors’ descriptions.

Defining chronic exposure as anything longer than 24 h complicates the analysis, as this category includes a wide range of durations, making it hard to differentiate between intermediate and prolonged effects. Additionally, temporary changes during the exposure period were not considered, and the long-term impact of CORT on overall organismal health was not captured.

Variability in antibody use across studies adds further complexity, including differences in concentrations and manufacturers. Several studies also lacked antibody catalog numbers, which hinders reproducibility.

Lastly, this review did not conduct a formal quality assessment of the studies, which could affect confidence in the findings. These limitations emphasize the need for future research to standardize methodologies and explore less-studied regions and systems.

### 4.8. Directions for Future Research

This review identifies several areas for further investigation into corticosterone’s effects on AMPAR dynamics. Future studies should keep as many variables constant as possible, only varying factors such as AMPAR subunit, exposure duration, concentration, nervous system region, or experimental model. This systematic approach will help determine whether corticosterone’s effects are universal across the rodent nervous system or specific to certain regions and subunits.

Improving reproducibility and comparability requires stringent reporting and standardized animal handling protocols, including detailed documentation of environmental conditions. Initial research using neuronal cell cultures or isolated tissue models can establish baseline effects of corticosterone before progressing to live animal studies.

The current literature lacks sufficient studies applying corticosterone to isolated tissues. Such models can reduce variability and provide valuable insights into AMPAR dynamics. Future research should also explore the effects of CORT on GluR1, GluR2, and the less-studied GluR3 and GluR4 subunits to understand its broader impact on synaptic function.

Combining Western blotting, immunolabeling, and single-particle tracking may provide a more comprehensive understanding of CORT’s effects on AMPARs. Expanding the range of subjects—by including strain and sex differences in stress responses—could also improve the generalizability of findings.

Future research should examine GluR1 and GluR2 alongside the less-studied GluR3 and GluR4 subunits to better understand CORT’s effects on AMPARs. Investigating how these subunits are modulated by CORT exposure could provide insights into its broader effects on synaptic function. Ongoing research into AMPAR subunit trafficking, regulation, and composition remains critical for understanding stress’s effects.

While most studies have focused on the central nervous system, corticosterone acts through MR and GR receptors present in the peripheral nervous system [27,71]. In addition to the auditory system, examples include the retina, the gastrointestinal tract, and pain-sensing nociceptors in the spinal cord. Investigating how CORT affects AMPAR function in these systems would broaden our understanding of stress effects on neural circuits.

Exploring MR expression throughout the auditory pathway, beyond the inner ear, would also be valuable. Understanding where and how these receptors might influence AMPAR dynamics in peripheral and central nervous system regions may provide new insights into stress-related disorders, both auditory and otherwise. The findings of Whitehead et al. suggest that dexamethasone, a synthetic corticosteroid, may modulate AMPAR dynamics differently than corticosterone [80]; thus, researching dexamethasone’s effects across various nervous system regions could yield insights into stress-related disorders and the broader impacts of corticosteroid treatments.

Addressing these research gaps could enhance our understanding of stress effects across the nervous system and help develop targeted interventions for stress-related disorders, including tinnitus.

## 5. Conclusions

This scoping review summarizes research on the effects of corticosterone exposure on AMPA receptor dynamics in the rodent nervous system, particularly focusing on potential effects in the auditory system. Short-term CORT exposure generally increases AMPAR surface localization and mobility in neuronal cultures, but results from live animal models have shown significant variability due to differences in exposure conditions, species, and stress responses. Most studies have concentrated on the hippocampus, with some on the sensorimotor cortex, prefrontal cortex, amygdala, and visual cortex, yet no research has been conducted on AMPAR dynamics in the auditory system.

The studies identified in this review primarily focused on the hippocampus, with additional research on the sensorimotor cortex, prefrontal cortex, amygdala, and visual cortex. No research was identified on AMPAR dynamics in the auditory system. Further research is needed to investigate how corticosterone influences AMPARs in the auditory system, considering the potential role of MRs and GRs. Additionally, given the high variability observed in live animal models, further studies would benefit from standardized protocols and more straightforward reporting of exposure conditions. Investigating other AMPAR subunits, such as GluR3 and GluR4, and exploring other nervous system regions may also help to clarify CORT’s broader effects on AMPAR dynamics and its potential role in stress-related auditory disorders. Addressing these research gaps could enhance our understanding of the connection between stress and tinnitus, a relationship that has remained unexplained despite being acknowledged for centuries.

## Figures and Tables

**Table 1:** Regional outcomes of AMPAR protein levels and localization following corticosterone exposure.

Region	Outcomes After Acute CORT Exposure (<1 h)	Outcomes After Subchronic CORT Exposure (3 h)	Outcomes After Chronic CORT Exposure (>24 h)
Amygdala	—	—	↑ Protein levels; ↑ Surface localization
Hippocampus	↑↓ = Surface Localization; ↑ Mobility; ↓ Protein Levels	↑ Surface Localization; ↑ Mobility	↑↓ = Protein Levels
Prefrontal Cortex	↑ Surface localization	—	↓ Surface localization
Primary Motor Cortex	—	—	= Protein Levels
Sensorimotor Cortex	= Protein Levels	—	—
Visual Cortex	↑ Surface Localization; ↑ Mobility	—	—
Auditory system	—	—	—

Summary of AMPAR protein level and localization outcomes across brain regions following corticosterone (CORT) exposure. Exposure durations were categorized by us as acute (<1 h), subchronic (1–6 h), and chronic (>6 h). Outcomes are represented as follows: ↑ indicates an increase, ↓ a decrease, = no change, and ↑↓ = mixed results (a combination of increases, decreases, and no changes across experiments). “—” indicates no experiments were conducted on this condition in the specified region.

## Data Availability

This study extracted and synthesized data from published studies; no new data were generated. The extracted data are available in the Supplementary Materials (Table S1: Extracted_Data). Further inquiries can be directed to the corresponding author.

## Supplementary Materials

The following supporting information can be downloaded at: https://www.mdpi.com/article/10.3390/brainsci15020110/s1, Table S1: S1_Extracted_Data; Table S2: S2_Experimental_Details_and_Outcomes.
