# Supplementary material for: A Scoping Review of Corticosterone-Induced Changes in Ionotropic Glutamate Receptor Levels and Localization in the Rodent Brain: Implications for the Auditory System
Source: Brain Sci. 2025 Jan 24;15(2):110. doi: 10.3390/brainsci15020110 (PMC11852854; doi:10.3390/brainsci15020110)
Supplement: Supplementary file 1 [file brainsci-15-00110-s001.zip › S2_Experimental_Details_and_Outcomes.pdf]

Supplemental Table 2. Experimental details and extracted outcomes of corticosterone exposure on AMPARs. The respective references are sorted in alphabetical order.

First Author and Year	Experimental Model	Animal	Age	Area	CORT exposure duration	CORT concentration	Time from CORT exposure to reading	Measuring method	Main extracted findings
Chen, 2021	live animal	C57BL/6N mice, M+F	8-16 weeks	visual cortex	1 IP injection	5 mg/kg	1, 2, and 3 h post-injection	FRAP	SEP-GluA1 fluorescence recovery significantly increased to 90% 3 h following injection of CORT, suggesting that CORT injection causes a shift from a near equal split between mobile and immobile pools to an almost entirely mobile pool of GluA1-containing receptors within cortical synapses
Choi, 2018	live animal	ICR mice, M	7 weeks	hippocampus	1 IP injection	10 mg/kg	Not specified	WB, IC	Exposure to CORT showed reduced trafficking of AMPAR 1/2 into the synapse due to microtubule destabilization
Groc, 2008	cell culture	hippocampal cultured neurons from Sprague-Dawley rats	neuronal cells from E18 Sprague-Dawley rats; neuronal cultures 14-21 DIV	hippocampus	20 min	100 nM	1 - 150 min	IF	Corticosterone triggers time-dependent increases in GluR2-AMPAR surface mobility and synaptic surface GluR2 content. The peak stimulatory effects of corticosterone on surface GluR2-AMPAR mobility and relative synaptic content were observed at 150 min after application.
Kula, 2016	live animal	Wistar rats, M	5-6 weeks	primary motor cortex M1	SC injections twice daily for 7 days,	10 mg/kg	Tissue collection 2, 4 and 7 days after the last CORT administration	WB	Corticosterone did not influence the protein levels of GluA1 and GluA2 subunits

					or a single SC injection				
Li, 2019	live animal	Sprague-Dawley rats, M	3 and 9 weeks	hippocampus	IP injections once daily for 21 days	5 mg/kg	Tissue collection 48 h or four weeks after last injection	WB	In adolescents, both the GluA1 and GluA2 subunits were significantly upregulated by CORT treatment. GluA3 or GluA4 expression levels remained unchanged. No significant changes were observed for the GluR1, 2, 3 or 4 AMPA receptor subunits following CORT treatment in adults.
Li, 2021	cell culture	Sprague–Dawley rats	neuronal cells from P0–1 Sprague–Dawley rats; neuronal cultures 8–10 days	amygdala	24 h	50 mM	Overnight*	IC	CORT significantly enhanced the fluorescence intensity of GluA1-positive neurons
Liu, 2010	cell culture	Strain of rat not stated**	neuronal cells from E18 rats; neuronal cultures DIV 20	prefrontal cortex	30 min	100 nM	Experiment 1: 1.5 – 4 h Experiment 2: 2h	IC	Corticosterone profoundly increased surface GluR1 cluster density
Martin, 2009	cell culture	Strain of rat not stated**	neuronal cells from E18 rats; neuronal cultures DIV 13-20	hippocampus	3 h	100 nM	60 - 180 min	IC, FRAP	Both GluR1 and GluR2 surface expression are increased by corticosterone, but GluR2 is more sensitive and increases to a greater extent than GluR1. No change in GluR2 after 1 h of corticosterone but pronounced effects after 3 h suggestive of a mechanism involving GR-mediated transcriptional regulation. FRAP of SEP–GluR2 in hippocampal neurons shows that

									corticosterone treatment alters AMPAR lateral diffusion. Corticosterone mobilizes normally synaptically anchored surface expressed AMPARs.
Martisova, 2011	live animal	Wistar rats, M	12 weeks	hippocampus	35 days of SC implanted pellets	18 mg/kg	Tissue collection 35 days after pellet implantation	WB	GluR1 and GluR2/3 expression was decreased in chronic treatment with corticosterone. GluR4 increased.
Monsey, 2014	live animal	Sprague-Dawley rats, M	12 weeks	hippocampus and amygdala	Experiment 1: 14 days in drinking water. Experiment 2: additional 6 days of CORT titration (25 mg/ml for 3 days,12.5 mg/ml for 3 days) + 14 day 'wash-out' period	50 mg/ml drinking water	Experiment 1: tissue collection 0 days after the last CORT administration. Experiment 2: tissue collection 14 days after the last CORT administration	WB	Chronic corticosterone exposure resulted in an increase in GluR1 protein expression in the lateral amygdala. The enhanced expression of GluR1 persisted following the 14 day recovery period. Chronic corticosterone exposure resulted in a decrease in GluR1 protein expression in the hippocampal area CA3. The enhanced expression did not persist following the 14 day recovery period.
Sarabdjitsingh, 2014	cell culture	Sprague–Dawley rats	neuronal cells from E18 Sprague-Dawley rats; no data on DIV before CORT exposure	hippocampus	one pulse for 10 min or two consecutive pulses for 10 min with an interval of 60 min	100 nM	60 - 120 min	SPT	A single CORT pulse significantly increased the surface diffusion of GluA2–AMPAR. The introduction of a second pulse of CORT 60 min after the first abolished this effect.

Sarabdjitsingh, 2016	cell culture	Sprague-Dawley rats	neuronal cells from E18 Sprague-Dawley rats; no data on DIV before CORT exposure (14-21 DIV before GluA2-tracking)	hippocampus	four 10 min pulses with intervals of 60 min	100 nM	5 - 240 min	SPT	GluA2-AMPAR surface trafficking in hippocampal neurons is particularly responsive to the first pulse of corticosterone, less consistently to the 2nd and 3rd pulse and insensitive to the 4th pulse
Thacker, 2022	isolated tissue	Sprague-Dawley rats, M	6 weeks	sensorimotor cortex	30 min	200 nM	Not specified	WB	The relative presence of GluA1/GluA2 at the synaptic surface remained unchanged following treatment.
Xiong, 2015	cell culture	Wistar rats	neuronal cells from E18 rats; neuronal cultures 13-20 DIV	hippocampus	3 h	100 nM	Overnight*	IC, FRAP	Corticosterone increased surface expression of GluA1 and GluA2 AMPAR subunits. Corticosterone increased the mobile fraction of GluA2-containing AMPAR.
Yuen, 2011	cell culture	Sprague-Dawley rats	neuronal cells from E18 rats; neuronal cultures 24-30 DIV	prefrontal cortex	20 min	100 nM	60 - 240 min	IC	Corticosterone treatment induced a significant increase of synaptic GluR1 cluster density

*As stated in the paper. **The authors refer the reader to previous publications. Abbreviations: CORT: Corticosterone, DIV: Days in vitro, E: Embryonic day, F: Female, FRAP: Fluorescence Recovery after Photobleaching, IF: Immunofluorescence, IC: Immunocytochemistry, IP: Intraperitoneal, M: Male, P: Postnatal day, SC: Subcutaneous, SPT: Single Particle Tracking, WB: Western Blotting.
